# Small-molecule MDM2 antagonists attenuate the senescence-associated secretory phenotype

**DOI:** 10.1038/s41598-018-20000-4

**Published:** 2018-02-05

**Authors:** Christopher D. Wiley, Nicholas Schaum, Fatouma Alimirah, Jose Alberto Lopez-Dominguez, Arturo V. Orjalo, Gary Scott, Pierre-Yves Desprez, Christopher Benz, Albert R. Davalos, Judith Campisi

**Affiliations:** 1 Buck Institute for Research on Aging, 8001 Redwood Boulevard, Novato, CA 94945 USA; 2 Institute for Stem Cell Biology and Regenerative Medicine, Stanford University, School of Medicine, 1265 Welch Road, Stanford, CA, 94305 USA; 3 California Pacific Medical Center, Research Institute, 475 Brannan Street, San Francisco, CA 94107 USA; 4 Lawrence Berkeley National Laboratory, 1 Cyclotron Road, Berkeley, CA 94720 USA

## Abstract

Processes that have been linked to aging and cancer include an inflammatory milieu driven by senescent cells. Senescent cells lose the ability to divide, essentially irreversibly, and secrete numerous proteases, cytokines and growth factors, termed the senescence-associated secretory phenotype (SASP). Senescent cells that lack p53 tumor suppressor function show an exaggerated SASP, suggesting the SASP is negatively controlled by p53. Here, we show that increased p53 activity caused by small molecule inhibitors of MDM2, which promotes p53 degradation, reduces inflammatory cytokine production by senescent cells. Upon treatment with the MDM2 inhibitors nutlin-3a or MI-63, human cells acquired a senescence-like growth arrest, but the arrest was reversible. Importantly, the inhibitors reduced expression of the signature SASP factors IL-6 and IL-1α by cells made senescent by genotoxic stimuli, and suppressed the ability of senescent fibroblasts to stimulate breast cancer cell aggressiveness. Our findings suggest that MDM2 inhibitors could reduce cancer progression in part by reducing the pro-inflammatory environment created by senescent cells.

## Introduction

Cancer poses a major challenge to the longevity of mammals, and age is the largest risk factor for developing this disease^1^. Unlike many age-related pathologies, which are characterized by degeneration and loss of cell function, tumor cells must acquire new and aberrant functions to progress to deadly disease. Because persistent inflammation can trigger both degenerative diseases and cancer, an inflammatory tissue environment may link these pathologies^1^. One of the common features of aging is low-level chronic inflammation, termed sterile inflammation or “inflammaging”^2,3^. Even though all the sources of inflammaging are unclear, it likely derives at least partly from senescent cells^4^.

Cellular senescence can suppress tumorigenesis by halting the proliferation of pre-malignant cells^5,6^. Mammalian cells that are mitotically competent undergo senescence in response to stressful stimuli, including disrupted chromatin, DNA damage, strong mitogenic signals (e.g., activated oncogenes) and mitochondrial dysfunction^7,8^. Along with the permanent cell cycle arrest induced by the p53 and p16^INK4a^ tumor suppressors^9–11^, an important feature of senescent cells is the secretion of a myriad of biologically active factors, termed the senescence-associated secretory phenotype (SASP)^12^.

The SASP is similar between mice and humans^13–17^, and comprises inflammatory cytokines such as IL-6 and IL-8^18^. The SASP can disrupt the surrounding microenvironment and normal cell functions, and stimulate malignant phenotypes in nearby cells^13–15^. Senescent cells can also promote tumor growth in mice^16–19^. Because senescent cells increase with age^17–19^ and are frequently found within hyperplastic and degenerative tissues^20,21^, the SASP may be a major cause of inflammaging^22–25^. Compounds that modulate the SASP hold promise for ameliorating a number of diseases of aging, including cancer.

Nutlins were originally identified as potent small molecules that inhibit the interaction between p53 and MDM2, which promote p53 degradation^5,6,26^. Nutlin therefore stabilizes p53, thereby promoting the apoptotic death of cancer cells. Importantly, in cancer cells, nutlin-3a inhibits the activity of NF-κB, a potent transcriptional stimulator of genes encoding inflammatory cytokines, in a p53-dependent manner^27,28^. Thus, nutlin-3a is a potential anti-cancer drug that could simultaneously trigger p53 activation and NF-κB suppression. Moreover, loss of p53 impairs the repression of NF-κB target genes by glucocorticoids, and stabilization of p53 by nutlin-3a enhances the repression of NF-κB by the glucocorticoid receptor^29^. The clinical importance of small-molecule MDM2 inhibitors like nutlin-3a spurred the discovery of similar compounds, such as MI-63, which are more efficient inhibitors of the MDM2-p53 interaction^30^.

MDM2-p53 interaction antagonists can have paradoxical results. While inducing cell cycle arrest, high p53 activity can also suppress the senescence growth arrest, thus causing quiescence. Indeed, nutlin-3a was shown to suppress p21-induced senescence and convert senescence into quiescence^31^, a reversible growth arrested state. In another study, however, nutlin-3a reduced expression of inhibitor of growth 2 (ING2), increased expression of several microRNAs, and triggered cellular senescence^32^.

To understand these conflicting results, we investigated the effects of small-molecule MDM2-p53 interaction antagonists on senescent phenotypes, including the SASP, of primary human fibroblasts and epithelial cells. We used nutlin-3a, as well as the non-peptide small molecule inhibitor of MDM2, MI-63^33^. We compared these compounds for their ability to induce a growth-arrested state, whether quiescence or senescence, in human cells, and evaluated their ability to modulate the SASP. We found that both compounds trigger selected markers of a senescent-like state, but the growth arrest was reversible, and both significantly suppressed the SASP, suggesting potential utility as therapeutic agents.

## Results

### Effects of nutlin-3a and MI-63 on senescence phenotypes

**Figure 1:**
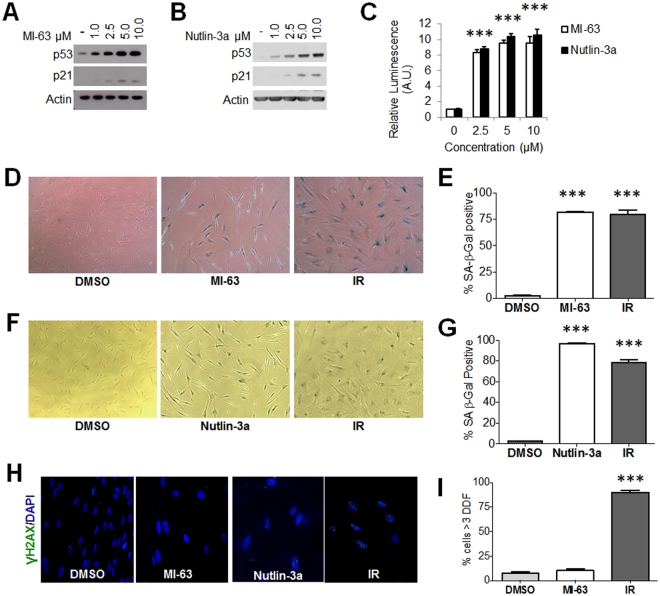
MDM2 inhibitors induce a senescence-like state. (**A**,**B**) HCA2 fibroblasts were treated using the indicated concentrations of MI-63 (**A**) or nutlin-3a (**B**). p53 and p21 levels were analyzed by western blotting. Actin levels served as a loading control. (**C**) IMR-90 fibroblasts were transduced with a p53 luciferase reporter and treated with MI-63 or nutlin-3a. Extracts were prepared and analyzed by luminometry. (**D**,**E**) HCA2 cells were treated with MI-63 or induced to senesce by 10 Gy IR. SA-β-gal staining is shown in (**D**) and percentage of positive cells is shown in (**E**). (**F**,**G**) HCA2 cells were treated with nutlin-3a or induced to senesce by 10 Gy IR. SA-β-gal staining is shown in (**F**) and percentage of positive cells is shown in (**G**). (**H-I**) HCA2 cells were irradiated (IR) or treated with MI-63 or nutlin-3a, and immunostained for γH2AX. Representative images are shown in (**H**) and percentage of cells with >3 γH2AX nuclear foci is shown in (**I**). Data are representative of two independent experiments. ***p < 0.001 by 1-way ANOVA.

Small-molecules that inhibit the p53-MDM2 interaction stabilize and often activate p53^34^. We confirmed that MI-63 and nutlin-3a increased protein levels of p53 and its transcriptional target p21 in a dose-dependent fashion in HCA2 primary human fibroblasts (Fig. 1A,B). To measure p53 activity, we transduced the cells with a lentiviral p53-reporter construct and measured reporter (luciferase) activity (Fig. 1C). Both compounds stimulated p53 activity at similar doses (2.5–5 μM).

A previous report suggested that nutlin-3a induces senescence^27^. Indeed, nutlin-3a and MI-63 induced the widely used senescence-associated beta-galactosidase (SA-β-gal) activity^35^, similar to the extent induced by 10 Gy ionizing radiation (IR) (Fig. 1D–G). In contrast to IR, however, nutlin-3a- and MI-63-treated cells had very few nuclear foci containing γH2AX, a marker of DNA double strand breaks and DNA damage signaling^20^ (Fig. 1H,I). Likewise, a previous report showed that MDM2-p53 interaction antagonists can cause cancer cells to undergo apoptosis^36^, a process to which senescent cells are resistant^37^. To determine how normal cells respond, we treated IMR-90 primary human fibroblasts with MI-63 or nutlin-3a and used immunofluorescence to measure cleaved (active) caspase-3, which executes both the intrinsic and extrinsic apoptotic pathways (Figure S1A,B). Neither compound significantly increased caspase-3 activation in contrast to 1 µM staurosporine, a known inducer of apoptosis.

We also measured loss of HMGB1 from the nucleus and its secretion, another common feature of senescent cells^26^. After a 24 h treatment with MI-63, HMGB1 left the nucleus, as determined by immunostaining (Figure S2A), and was secreted, as determined by ELISAs of conditioned media (Figure S2B). However, after 72 h, HMGB1 nuclear staining increased relative to cells treated for 24 h, and HMGB1 secretion declined. These findings suggest the senescence-like state may not be permanent. Together, the results show that nutlin-3a and MI-63 stabilize and activate p53, as expected, and also induce phenotypic features of senescence in normal human cells.

### MDM2-p53 interaction inhibitors induce a reversible growth arrest

**Figure 2:**
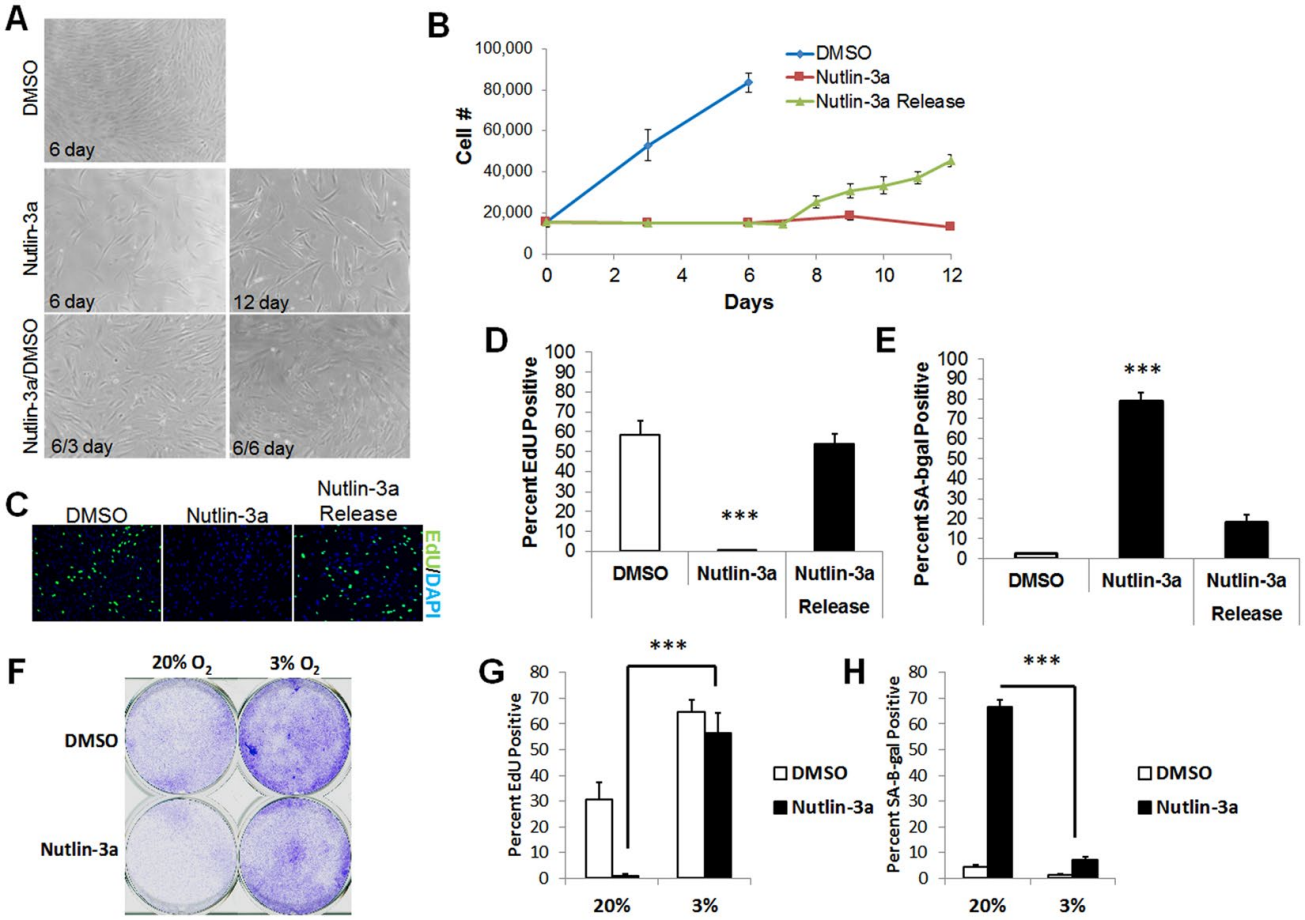
Reversibility of MDM2 antagonist-induced senescence. (**A**,**B**) Proliferating HCA2 fibroblasts were treated with DMSO or nutlin-3a, and cell number measured over the ensuing 6 d, at which point nutlin-3a was either replaced or exchanged for DMSO (Release) for an additional 6 d. Representative images of the cell cultures are shown in (**A**) and cell numbers are shown in (**B**). (**C**–**E**) IMR-90 fibroblasts were treated as in (**A**,**B**), and proliferation measured by EdU incorporation (24 h labeling period). (**C**) Representative EdU labeling images. (**D**) Percentage of EdU-positive cells. (**E**) Percentage of cells that stained positive for SA-β-gal. (**F**,**H**) IMR-90 cells were cultured in 3% or 20% O_2_, and DMSO or nutlin-3a for 6. Cells were then reseeded in drug-free medium and cultured for additional 6 d, followed by analysis for (**F**) clonogenic growth, (**G**) EdU labeling (24 h), and (**H**) SA-β-gal. Data are representative of three independent experiments. ***p < 0.001 by 1-way ANOVA or t-test.

Nutlin-3a was shown to induce a p53-dependent senescence growth arrest in mouse fibroblasts^38^. To assess its effect on human fibroblast proliferation, we treated HCA2 cells with 5 μM nutlin-3a, and measured cell number over a 6 to 12 day interval (Fig. 2A,B). After 6 days, vehicle-treated cells increased in number approximately 4-fold, whereas cells treated with nutlin-3a failed to proliferate. However, upon nutlin-3a removal after 6 days, cells resumed proliferation, as measured by cell number (Fig. 2A,B) and EdU incorporation (Fig. 2C,D). IMR-90 cells behaved similarly (Fig. 2D), and also showed a significant decrease in SA-β-gal activity (<10% positive) after nutlin-3a removal (Fig. 2E).

Our results indicating reversibility of the nutlin-3a growth arrest are apparently at odds with a previous study indicating an irreversible senescence growth arrest^35^. We speculated that oxygen levels might determine whether the nutlin-3a-induced growth arrest is permanent. We routinely culture cells in 3% oxygen, which is closer to normoxia for many cells, whereas culture in atmospheric oxygen, which is hyperoxic, is more common^33^. Indeed, IMR-90 cells cultured in atmospheric oxygen failed to regrow after nutlin-3a was removed (Fig. 2F,G). However, the same cells cultured in 3% oxygen resumed growth after nutlin-3a removal, as evidenced by increased EdU incorporation, reduced SA-β-gal staining and increased colony formation (Fig. 2F–H). These data show that nutlin-3a induces a senescent-like phenotype in human fibroblasts, but, depending on the oxygen concentration, the phenotype can be reversible.

### MDM2-p53 interaction antagonists attenuate the SASP

**Figure 3:**
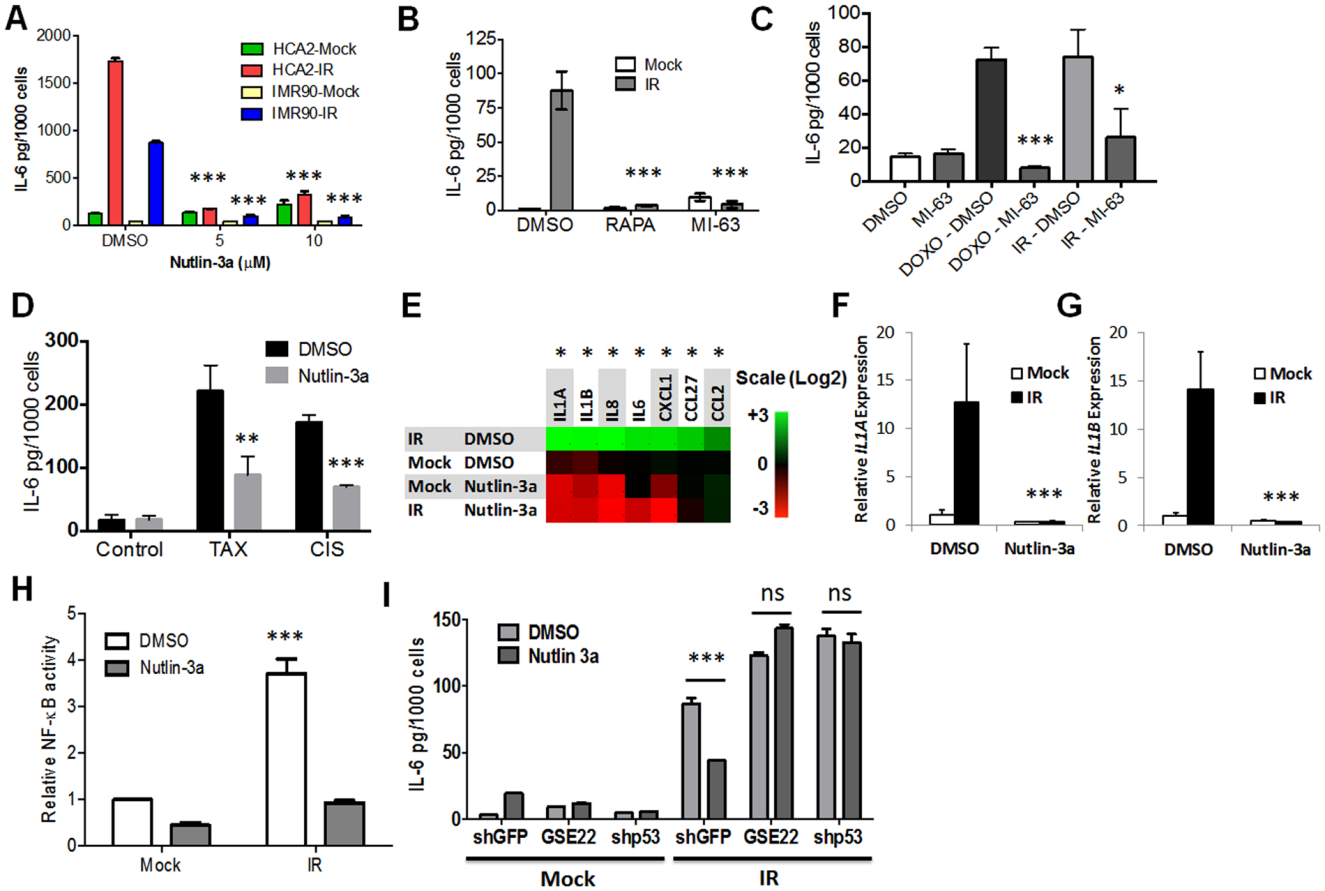
MDM2 antagonist attenuates the SASP. (**A**) IL-6 ELISA using conditioned media (CM) from HCA2 and IMR-90 fibroblasts that were induced to senesce by IR and treated with DMSO or nutlin-3a at two concentrations. (**B**) IL-6 in CM from IMR-90 cells (mock or IR) after treatment with DMSO, rapamycin (RAPA) or MI-63. (**C**,**D**) IMR-90 cells were treated for 24 h with the chemotherapeutic agents doxorubicin (DOXO) (**C**), taxol or cisplatin (**D**), and IL-6 secretion measured by ELISA in cells cultured with MI-63 (**C**) or nutlin-3a (**D**) for 24 h following removal of the drugs. (**E**) Heat map indicating gene expression of several SASP factors in IMR-90 fibroblasts (mock or IR) treated DMSO or nutlin-3a. (**F**,**G**) Relative expression of (**F**) *IL-1α* and (**G**) *IL-1β* in control or irradiated (IR) fibroblasts cultured with nutlin-3a or DMSO. (**H**) HCA2 cells were transduced with a lentiviral NF-κB luciferase reporter construct, and luciferase activity in DMSO- or nutlin-3a-treated (mock or IR) cells was measured. (**I**) HCA2 fibroblasts were transduced with lentiviruses expressing either control (shGFP) or p53-targeted shRNAs (shp53) or a p53 genetic suppressor element (GSE22) and treated with DMSO or nutlin-3a for 24 h following mock or X-irradiation (IR). IL-6 secretion was measured by ELISA. Data are representative of three independent experiments. *p < 0.05, ***p < 0.001 by 2-way ANOVA.

Senescent cells secrete a plethora of biologically active molecules, called the senescence-associated secretory phenotype (SASP)^39^, which promote malignant and aging phenotypes^14,18^. Since MDM2-p53 interaction antagonists were developed as cancer therapeutics, we evaluated their effects on interleukin-6 (IL-6), a SASP factor that can promote malignant phenotypes^5,6,9,26^. Continuous treatment with nutlin-3a significantly lowered IL-6 secretion by IMR-90 and HCA2 fibroblasts induced to senesce by IR (Fig. 3A). In IMR-90 cells, MI-63 was as potent as rapamycin, which suppresses IL-6 expression and the pro-malignant effects of the SASP^14,18^ (Fig. 3B). In mammary epithelial cells MCF10A (Figure S3A) and 184A1a (Figure S3B), MI-63 was more potent at reducing IL-6 secretion than rapamycin.

IL-6 attenuation was observed in IMR-90 fibroblasts treated with MI-63 for 5 or 6 days prior to analysis (Figure S3C). However, if MI-63 was added after the SASP developed (1 day before analysis), no attenuation was observed (Figure S3C). We hypothesized that once the SASP develops, cells may require longer exposures to MDM2-antagonists before IL-6 levels decline. To test this notion, we treated cells for 7 days with nutlin-3a either immediately following IR (IR-7D) or after waiting 7 days for the SASP to develop (IR-14D) (Figure S3D). Nutlin-3a diminished, but did not completely eliminate, IL-6 expression in cells that already expressed a SASP (Figure S3D), suggesting partial efficacy in reducing effects of the SASP.

While MDM2 inhibitors are currently in clinical trials as primary cancer therapeutics, the loss of senescence-associated IL-6 secretion suggests they may also be useful as adjuvants to senescence-inducing chemotherapies. We treated IMR-90 cells with the genotoxic chemotherapeutic agents doxorubicin (DOXO), taxol (TAX) or cisplatin (CIS), and measured IL-6 secretion in the presence or absence of an MDM2 inhibitor. Each agent induced DNA damage foci (DDF) (Figure S3E,F) and IL-6 secretion (Fig. 3C,D), as expected^40^. Significantly, MDM2 antagonists effectively attenuated the IL-6 secretion resulting from DOXO- (Fig. 3C), as well as TAX- and CIS- (Fig. 3D), induced senescence. MDM2 antagonists also reduced the SASP resulting from RAS-induced (Figure S3G) and replicative (Figure S3H) senescence. These results suggest an additional target (the SASP of senescent cells) for clinical applications of small-molecule MDM2 inhibitors beyond primary tumor cells.

To determine whether inhibition of the SASP extended beyond IL-6, we measured the expression of several SASP mRNAs by quantitative PCR in control (Mock) or senescent (IR) cells continuously treated with DMSO or nutlin-3a (Fig. 3E). Among the genes significantly down-regulated by nutlin-3a were *IL-1α* and *IL-1β* (Fig. 3F,G), signaling through which regulates several other SASP genes^41^. A longer list of SASP factors down-regulated by MI-63 is presented in Table S1.

Because the SASP appears gradually and depends on IL-1α activation of NF-κB^42^, we transduced HCA2 cells with a lentiviral NF-κB-luciferase reporter, and assessed relative NF-κB transcription activity in DMSO- or nutlin-3a-treated cells by luciferase activity (Fig. 3H). Nutlin-3a significantly reduced NF-κB activity, suggesting a decrease in NF-κB activation mediates the reduced expression of SASP factors at the transcriptional level.

To determine whether nutlin-3a lowers SASP gene expression through activation of p53, we abrogated p53 activity by either lentiviral-delivered shRNA depletion (shp53)^41,42^ or overexpression of a p53 genetic suppressor element peptide (GSE22)^43^ (Fig. 3I). Upon loss of p53 activity, nutlin-3a no longer lowered IL-6 secretion levels, indicating that p53 is indeed required for MDM2-antagonist mediated suppression of the SASP. Thus, the two small molecule MDM2-antagonists we analyzed potently suppressed the secretory phenotype of fibroblasts induced to senesce by various means.

### MDM2-inhibitors repress the ability of senescent cells to stimulate cancer cell aggressiveness

**Figure 4:**
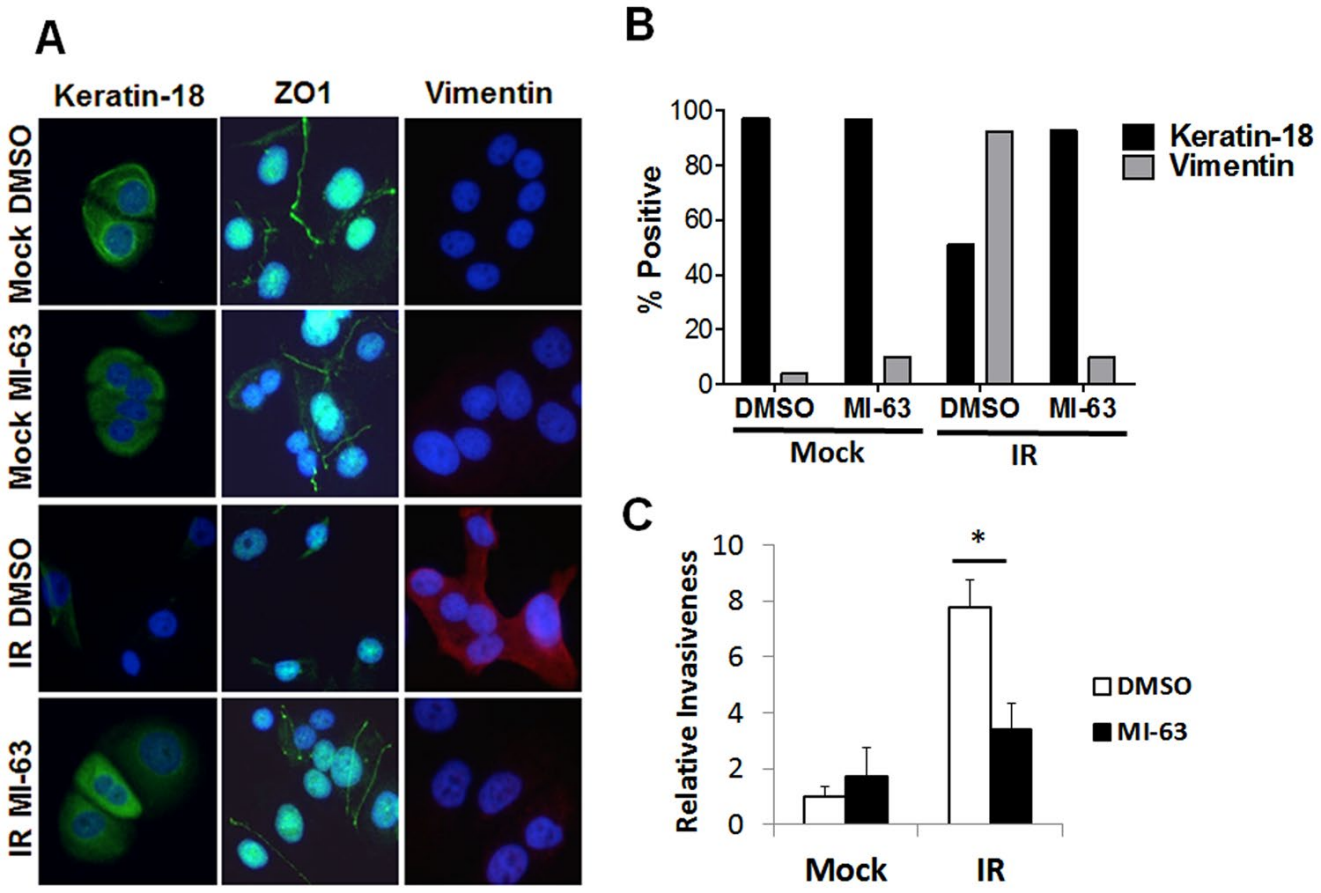
Effect of MDM2 inhibitors on ability of senescent cells to stimulate cancer cell aggressiveness. (**A**,**B**) ZR75-1 human breast cancer cells were cultured in the presence of CM from DMSO- or MI-63-treated (mock or IR) HCA2 fibroblasts for 3 d, and immunostained for ZO-1, cytokeratin 18 or vimentin. (**A**) Representative images of the staining. (**B**) Quantification of staining (percentage of positive cells). (**C**) CM described in (**A**) was assayed for ability to stimulate ZR75-1 cancer cells to invade a basement membrane. Invasion stimulated by CM from DMSO-treated mock fibroblasts was given a value of one, and other conditions were normalized to this value. Data are means of 4 independent experiments (*p < 0.05, t-test). Error bars indicate standard deviation of the mean.

We showed that conditioned media (CM) from senescent cells promote cancer cell aggressiveness by inducing an epithelial to mesenchyme transition (EMT) in non-aggressive breast cancer cell lines such as ZR75-1^44^. The EMT is characterized by diminished expression of the tight junction protein ZO-1 and the epithelial protein keratin 18. As expected, immunostaining showed that ZR75-1 cells cultured in CM from DMSO-treated senescent fibroblasts expressed the mesenchymal marker vimentin and little ZO-1 and keratin 18 (Fig. 4A,B). Importantly, ZR75-1 cells cultured in CM from MI-63-treated senescent fibroblasts showed increased expression of ZO-1 and keratin 18 and lower levels of vimentin (Fig. 4A,B). Consistent with the SASP mediating cancer cell aggressiveness, MI-63 suppressed the ability of senescent fibroblast CM to stimulate ZR75-1 cell invasiveness through a basement membrane mimetic (Fig. 4C).

Thus, small-molecule MDM2 antagonists can indirectly reduce the aggressive phenotype of breast cancer cells by suppressing the SASP of neighboring cells, suggesting an unexpected potential therapeutic benefit.

## Discussion

Cellular senescence suppresses tumorigenesis by preventing the proliferation of pre-malignant cells^19^. Many potentially oncogenic stimuli induce normal mammalian cells to senesce in culture and *in vivo*^7,8^. Senescent cells generally lose the ability to divide, essentially irreversibly, and secrete numerous cytokines, growth factors and proteases^7,8,45^. Several studies indicate that this senescence associated secretory phenotype (SASP) ironically can create a more permissive environment for tumorigenesis^14^. Thus, while chemotherapy-induced senescence of tumor cells might prevent tumor growth, the resulting SASP may fuel the proliferation of tumor cells not eradicated by chemotherapeutics.

The identification of signaling pathways utilized by cancer cells spurred the development of small molecules targeting these pathways^14,15,41,46^. Mutations in the p53 tumor suppressor protein occur in ~50 percent of cancers^31^. However, some cancers, such as neuroblastoma, rhabdomyosarcoma and acute myeloid leukemia, often retain wild type p53^47^, which led to the development of small molecules that increase p53 activity. Among these molecules are nutlin 3a and MI-63^48–50^. Glioblastoma cells, for example, respond to nutlin-3a by undergoing a p53-dependent cell cycle arrest with features of senescence, as well as undergoing apoptosis^51^. Moreover, nutlin-3a increased the response of glioma cells to radiation therapy^37^.

MDM2 inhibitors were reported to induce normal cells to undergo cellular senescence, as evidenced by absence of colony formation and SA-β-gal activity, following extended exposures^52^. We initially concluded that the MDM2 inhibitors induced “classic” senescence, including an irreversible proliferative arrest. The inhibitors induced SA-β-gal positivity and an initial relocalization and secretion of HMGB1 that was indistinguishable from senescence induced by, for example, IR. However, a more in depth analysis showed that many of the effects of both nutlin-3a and MI-63 were transient. We assessed colony formation, HMGB1 relocalization and SA-β-gal activity as well as the appearance of DDF and secretion of inflammatory cytokines. Several strains of primary fibroblasts lacked these hallmarks upon MDM2 antagonist treatment, and they resumed growth and lost SA-β-gal staining upon removal.

What might account for the differences between these observations? Previous studies cultured cells in atmospheric oxygen, while we culture cells in more physiological oxygen levels (3%). We indeed found that cells arrested growth irreversibly when treated with nutlin-3a in atmospheric oxygen. However, at lower oxygen levels, nutlin-3a treated cells resumed growth, incorporated EdU, formed colonies and retained nuclear HMGB1 after drug removal. We also speculate that MDM2 inhibitors increase p53 to a level sufficient to repress genes that regulate the SASP factors.

Several reports suggest that cells cultured in supraphysiological (atmospheric) oxygen experience diverse deleterious effects, including DNA damage and genomic instability. Importantly, reactive oxygen species are known to impact chromatin-modifying enzymes, which can lead to epigenetic changes in gene regulation^53^. Thus, we further speculate that cells cultured at supraphysiological oxygen levels experience chromatin changes that prevent the resumption of proliferation after removal of MDM2 inhibitors. Since tissues are rarely exposed to atmospheric oxygen levels^33,35^, MDM2 antagonists might be safer therapeutics than previous studies would suggest, as any growth arrest induced by these drugs *in vivo* is likely to be reversible.

In invasive pineal tumor cells, nutlin-mediated p53 restoration was effective at inducing senescence only when paired with DNA-damaging therapy, and even then was reversible upon nutlin-3a removal^39^. Likewise, our data highlight the complexities of the clinical potential of administering MDM2 inhibitors as an adjuvant during chemotherapy. We showed that genotoxic anti-cancer treatments (IR, doxorubicin, cisplatin and taxol) induce senescence and a robust SASP, as expected^40^. However, an MDM2 antagonist greatly attenuated this SASP. Following senescence-inducing treatments, the SASP requires time before showing maximal secretion of cytokines such as IL-6, with higher secretion 14 days after senescence induction compared to 7 days after induction. Our results indicate that MDM2 inhibitors act prior to establishment of the SASP. Once the SASP is fully engaged, a short exposure of MDM2 inhibitor failed to attenuate the secretory profile. These data suggest that MDM2 inhibitors suppress pathways that regulate initiation of the SASP. Once established, the SASP may require additional interventions to dampen secretion and/or longer exposure to MDM2 inhibitors.

By suppressing the secretion of SASP factors such as IL-6, the MDM2 inhibitors suppressed the ability of senescent human fibroblasts to stimulate breast cancer cell aggressiveness (invasiveness and an EMT). Thus, these inhibitors might not only limit the growth of p53-positive tumors, but might also limit the cancer-promoting effects of senescent cells generated by genotoxic chemotherapies. Beyond cancer, MDM2 inhibitors might also provide an effective and unexplored strategy to reduce some of the effects of senescent cells in driving aging and age-related pathologies.

## Methods

All methods were performed in accordance with the relevant guidelines and regulations.

### Cells and reagents

Human HCA2 foreskin fibroblasts (originally from O. Pereira-Smith, University of Texas Health Science Center, San Antonio, TX), and fetal lung fibroblasts IMR-90 and WI-38 (from the Coriell Cell Repository, Camden, NJ), were cultured in 3% O_2_ and 10% CO_2_ in Dulbecco’s modified Eagle’s media (DMEM), 10% fetal bovine serum and 100 U/ml streptomycin/penicillin as described^54^. Non-senescent cells had a 24 h EdU labeling index of >75%. Human mammary epithelial cells MCF10A and 184A1a were cultured in MEGM (Lonza), human breast cancer cells ZR75-1 were cultured in DMEM at ambient oxygen and 5% CO_2_, and all three were from the American Type Culture Collection. All cultures were mycoplasma free. Nutlin-3a was obtained from Sigma Aldrich and Sanofi-Aventis provided MI-63. We did not initiate cultures from human tissue samples for this project; only previously established cells were used. Based upon this information, Exempt Status was approved by the Buck Institute Institutional Review Board.

### Antibodies for western analyses

Primary antibodies used for western blotting were: anti-actin (Abcam), anti-p53 (DO-1, Santa Cruz) and anti-p21 (BD Biosciences). Secondary antibodies were: goat anti-mouse IgG HRP conjugate (BioRad), or goat anti-rabbit IgG HRP conjugate (BioRad).

### Virus production and infections

p53 shRNA and GSE22 were previously described^43,44^. 293FT packaging cells (Invitrogen) were used to generate lentiviruses.

### Induction of senescence and SA-β-gal staining

To induce senescence by IR, cells were cultured to confluence, irradiated (10 Gy) using an X-ray generator (X-ray Associates) and seeded at lower density. 7–10 d later, cells displayed an enlarged senescent morphology and expressed SA-β-gal^20,21^.

### Real-time PCR

RNA was isolated from cells using a commercial kit (Biolline) according to the manufacturer’s instructions. Real time PCR was done using the Roche Universal Probe Library (UPL) system on a Lightcycler 480 II according to manufacturer’s specifications.

### Western blotting

After cells were lysed in buffer (Cell Signaling Technology), lysates were sonicated (10 sec) and centrifuged. Samples were boiled and loaded on 4–12% gradient bis-tris SDS-polyacrylamide gels. Proteins were separated by electrophoresis, and transferred to PVDF membranes, which were blocked in TBST 5% milk at room temperature (RT) for 1 h. Membranes were probed overnight at 4 °C with primary antibodies, washed in TBST, incubated in secondary antibodies conjugated to horseradish peroxidase for 1 h at RT, and developed using Western detection substrate (GE Healthcare).

### Immunofluorescence

Cells in 4 or 8-well chamber slides were fixed in 4% formaldehyde (Ted Pella Inc.) for 10 min, permeabilized in PBS-0.5% Triton for 10 min, blocked for 60 min using 10% goat serum (Invitrogen), and incubated overnight with primary antibodies (in blocking buffer) all at 4 °C. Primary antibodies were rabbit anti-HMGB1 (Abcam), mouse anti-γH2AX (Millipore), rabbit anti-caspase-3 (Cell Signaling), mouse anti-ZO-1, mouse anti-keratin 18 (Santa Cruz Biotechnology), and rabbit anti-vimentin (NeoMarkers). Cells were washed, incubated with secondary antibodies (Alexa Fluor 488 goat anti-rabbit IgG or Alexa Fluor goat anti-mouse 594; Invitrogen) for 30 min at RT, and washed 3× in PBS. The final wash contained 0.1 mg/ml DAPI. We mounted slides in Vectashield (Vector Labs). Images were obtained using an Olympus BX20 fluorescence microscope and Spotfire software (Diagnostics Instruments), and processed with Photoshop CS (Adobe).

### Luciferase reporter assays

Cells were co-transduced with a lentiviral NF-κB luciferase reporter construct or p53-luciferase reporter construct (SA Biosciences), and a constitutively expressed renilla luciferase construct (SA Biosciences). Cells were lysed in passive lysis buffer (Promega), and transcription activity was determined by firefly luciferase activity normalized to renilla activity using a dual luciferase kit (Promega).

### EdU labeling

Cells (10^4^) were seeded on chamber slides or coverslips, washed after 24 h, and EdU-containing media was added for 24 h. Cells were fixed, permeabilized in 0.5% Triton X-100, and processed using the Click-iT® EdU Alexa Fluor® 488 HCS Assay (Invitrogen).

### Invasion assay

ZR75–1 cells (80,000 per well) were plated on Matrigel that coated the upper chambers of Transwells (BD Biosciences). The lower chambers contained conditioned media (CM) from non-senescent or senescent HCA2 cells previously treated with DMSO or MI-63, and washed prior to CM collection. After 18 h, cells present on the underside of the upper chamber filter were stained and counted.

### Elisa

CM were prepared by washing cells 3 times in PBS, then incubating in serum-free media for 24 h. CM were filtered and stored at −80 °C, and cell numbers were determined. AlphaLISA IL-6 Immunoassay (Perkin Elmer; AL223F) and HMGB1 ELISA (IBL, International) were performed as per the suppliers’ instructions. All data were normalized to cell number.

### Statistical analyses

Error bars on all graphs show the standard error of multiple independent measurements. Statistical analyses were done using Graphpad Prism software. Significance was determined using Student’s t-test or ANOVA, as appropriate.

### Data availability

All data generated or analyzed during this study are included in the published article and Supplementary Information files.

## Electronic supplementary material

Supplementary Information
