# Supplementary material for: Small-molecule MDM2 antagonists attenuate the senescence-associated secretory phenotype
Source: Sci Rep. 2018 Feb 5;8:2410. doi: 10.1038/s41598-018-20000-4 (PMC5799282; doi:10.1038/s41598-018-20000-4)
Supplement: Supplementary file 1 — Supplementary Information [file 41598_2018_20000_MOESM1_ESM.pdf]

Small-molecule MDM2 antagonists attenuate the senescence-associated secretory phenotype

Christopher D. Wiley, Nicholas Schaum, Fatouma Alimirah, Jose Alberto Lopez-Dominguez, Arturo V. Orjalo, Gary Scott, Pierre-Yves Desprez, Christopher Benz, Albert R. Davalos, and Judith Campisi

SUPPLEMENTARY FIGURE LEGENDS

Figure S1. (A-B) IMR-90 human fibroblasts were treated with DMSO, MI-63 or nutlin-3a for 48 h, or 1 μM Staurosporine for 4 h, and immunostained for cleaved caspase-3. (A), representative images. (B), percentage of cleaved caspase-3-positive cells. Data are representative of two independent experiments. ** = $p < 0.01$, 1-way ANOVA.

Figure S2. (A) IMR-90 human fibroblasts were immunostained for HMGB1 (red) and p53 (green) following a 24 or 72 h treatment with MI-63. (B) CM from treated cells was analyzed for HMGB1 by ELISA. Data are representative of two independent experiments. ** = $p < 0.05$, 1-way ANOVA.

Figure S3. (A-B) Human mammary epithelial MCF10A (A) and 184A1a (B) cells were induced to senesce by ionizing radiation (IR), and treated with either rapamycin (RAPA) or MI-63. (C) IMR-90 human fibroblasts induced to senesce by IR were cultured with DMSO, or continuously with 10 μM MI-63 for 1, 5 or 6 d after irradiation. ELISA was

used to measure IL-6 secretion. (D) HCA2 fibroblasts were irradiated and DMSO or nutlin-3a was added either immediately (IR-7D), or after 7 days (IR-14D). CM was collected 7 d later and analyzed for IL-6 by ELISA. (E-F) IMR-90 fibroblasts were treated with DMSO, 10 μM nutlin-3a (Nutlin), 250 nM doxorubucin (DOXO), 4 μM cisplatin (CIS) or 20 nM taxol (TAX) for 24 h. Cells were cultured in drug-free media for 6 d, stained with anti-γ-H2AX antibody (green) (E), and cells with >3 foci were quantified (F). (G) IMR-90 cells were induced to senesce upon RAS overexpression. Cells were cultured with DMSO or MI-63 for 72 h and IL-6 levels measured by ELISA. (H) IL-6 secretion was determined in WI-38 human fibroblasts induced to senesce upon replicative exhaustion and treated with DMSO or MI-63. Data are representative of two independent experiments. ** = $p < 0.01$, *** = $p < 0.001$, 1-way ANOVA.

Table S1. IMR-90 human fibroblasts were induced to senesce by ionizing radiation (IR) or doxorubucin. Cells cultured in DMSO or MI-63 were analyzed by multiplex ELISA for the indicated proteins. Shown is the straight fold-increase upon IR or doxorubicin treatment.

S1

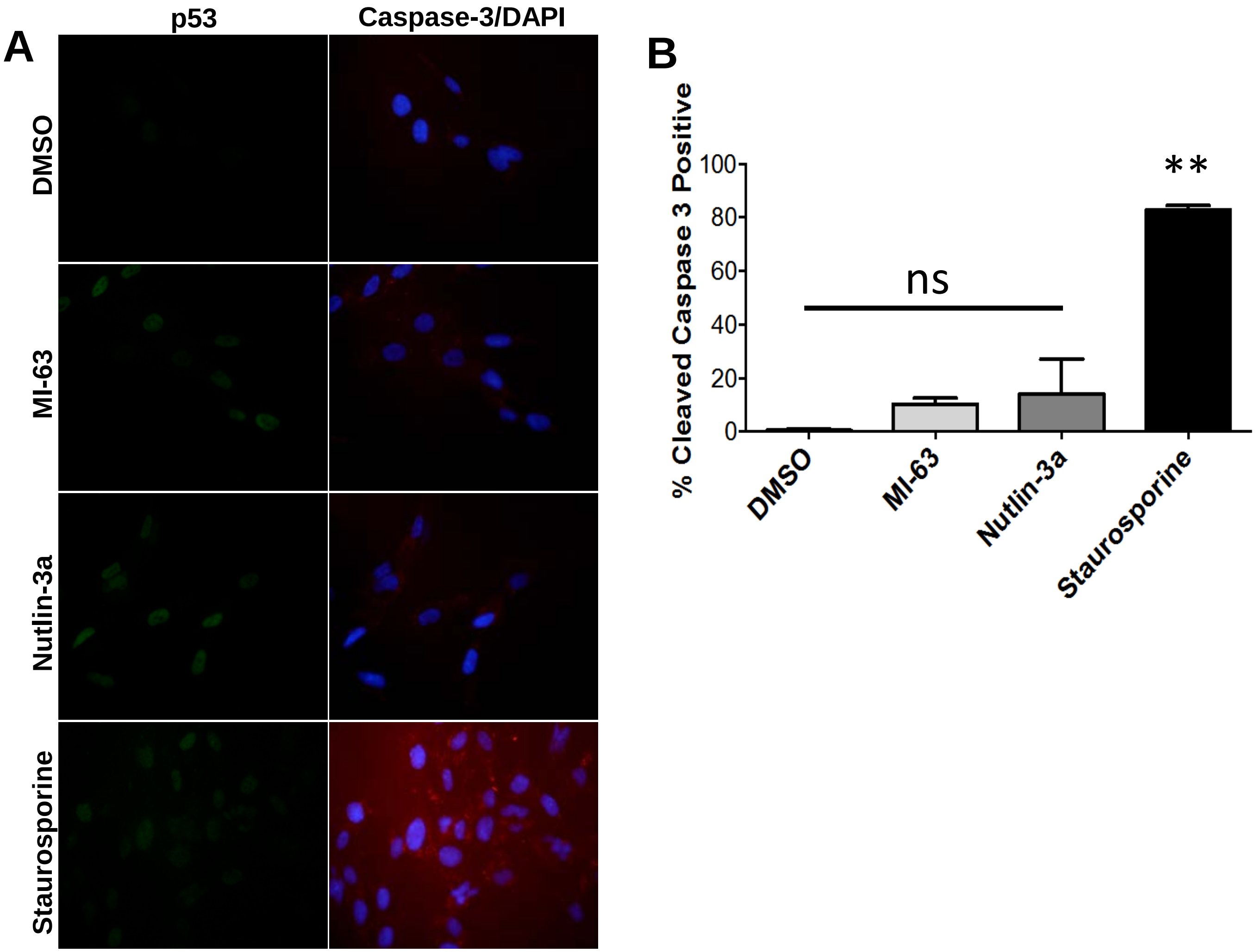
A
p53
Caspase-3/DAPI
DMSO
MI-63
Nutlin-3a
Staurosporine
B
% Cleaved Caspase 3 Positive
100
80
60
40
20
0
ns
**
DMSO
MI-63
Nutlin-3a
Staurosporine

S2

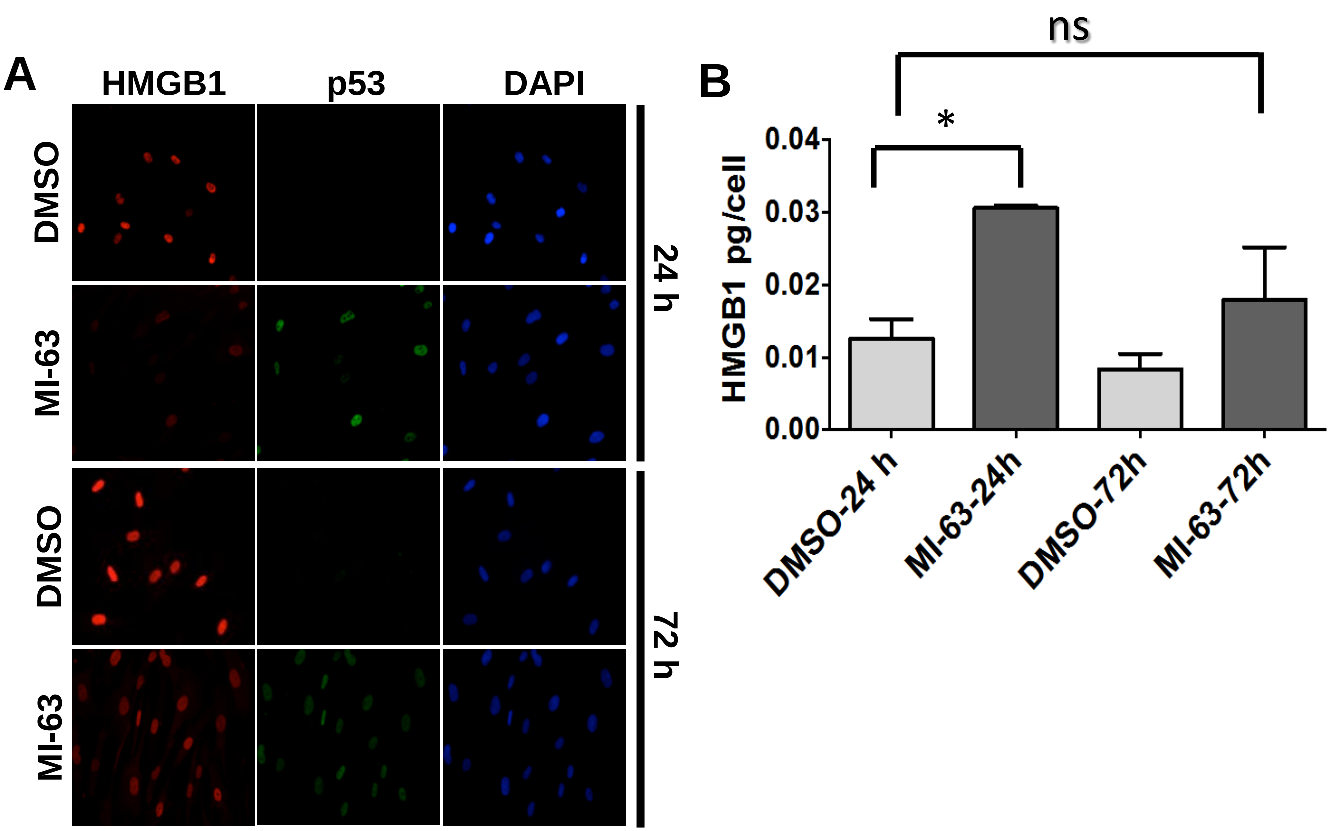
A
HMGB1
p53
DAPI
DMSO
MI-63
DMSO
MI-63
24 h
72 h
B
ns
*
HMGB1 pg/cell
0.04
0.03
0.02
0.01
0.00
DMSO-24 h
MI-63-24h
DMSO-72h
MI-63-72h

S3

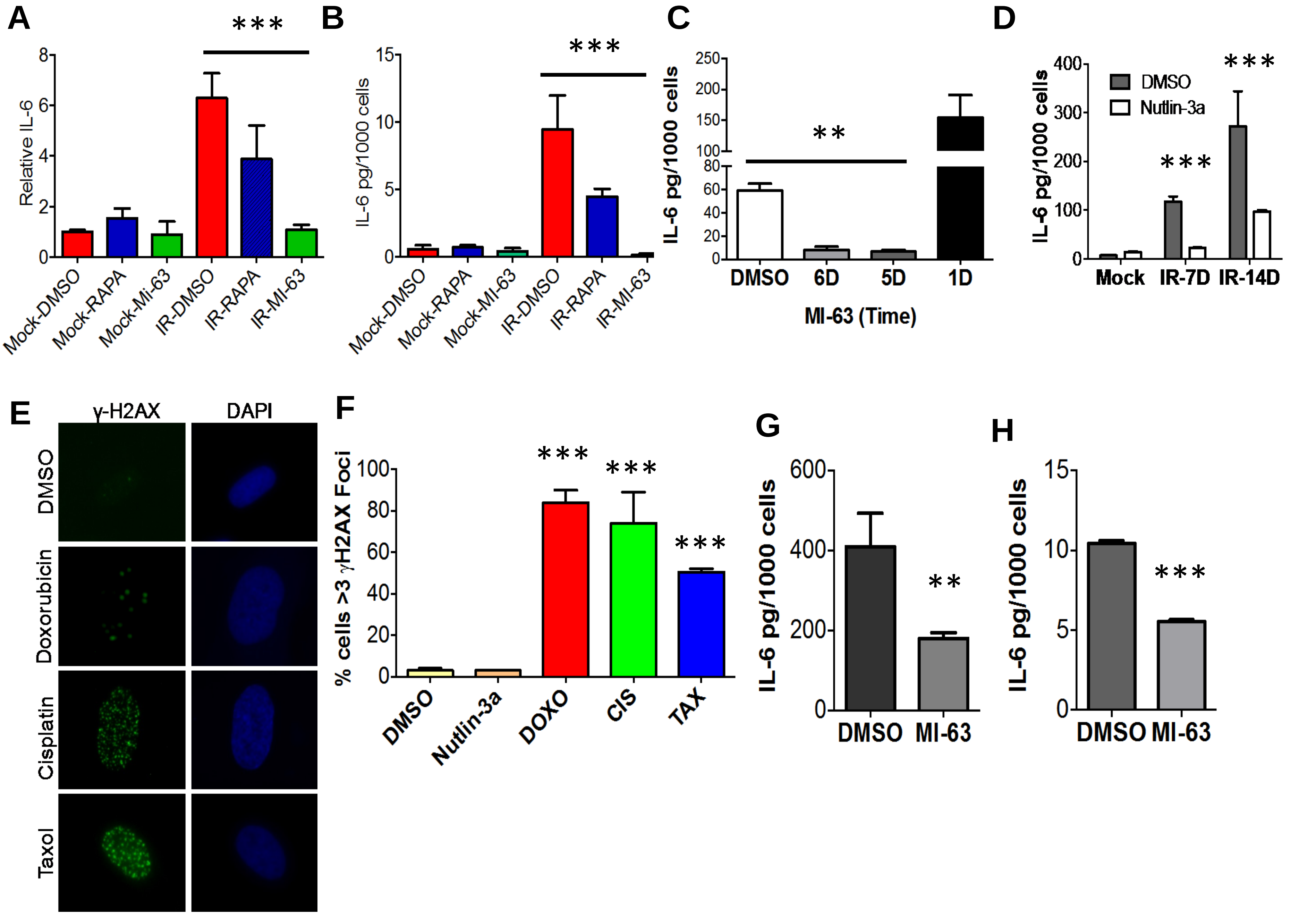

A
B
C
D
E
F
G
H
Relative IL-6
IL-6 pg/1000 cells
Mock-DMSO
Mock-RAPA
Mock-Mi-63
Mock-MI-63
IR-DMSO
IR-RAPA
IR-MI-63
DMSO
6D
5D
1D
MI-63 (Time)
Mock
IR-7D
IR-14D
Nutlin-3a
γ-H2AX
DAPI
Doxorubicin
Cisplatin
Taxol
% cells >3 γH2AX Foci
DOXO
CIS
TAX
MI-63

Table S1

	Mock		Doxorubicin		IR	
	DMSO	MI-63	DMSO	MI-63	DMSO	MI-63
IL6	1.0	1.0	174.2	1.0	242.6	1.0
IP10	1.0	1.0	62.2	1.0	50.8	1.0
VCAM1	1.0	1.0	48.3	1.0	24.8	1.0
IL7	1.0	0.5	25.6	0.5	25.3	0.5
LIF	1.0	0.7	13.7	0.5	10.3	1.5
ICAM1	1.0	0.4	12.4	0.4	7.1	0.4
HGF	1.0	1.0	11.1	0.5	4.0	0.9
TRAIL	1.0	1.0	12.5	1.0	2.5	1.0
MCP1	1.0	0.7	7.6	0.2	4.8	0.6
EOTAXIN	1.0	0.4	7.1	0.3	4.4	0.2
VEGF	1.0	0.5	5.9	0.2	4.8	0.6
IL8	1.0	0.1	4.8	0.2	6.7	0.0
RANTES	1.0	1.0	5.0	1.0	3.6	1.0
GMCSF	1.0	0.8	3.8	0.3	3.2	0.3
IL12P70	1.0	0.5	2.7	0.3	2.1	0.3
IL5	1.0	0.4	2.3	0.2	1.7	0.2
